# ACAT2 Is a Novel Negative Regulator of Pig Intramuscular Preadipocytes Differentiation

**DOI:** 10.3390/biom12020237

**Published:** 2022-01-31

**Authors:** Ye Tian, Yuelei Zhao, Wensai Yu, Sherif Melak, Yingfang Niu, Wei Wei, Lifan Zhang, Jie Chen

**Affiliations:** 1 Laboratory of Molecular Genetics and Animal Breeding, College of Animal Science and Technology, Nanjing Agricultural University, Nanjing 210095, China; hollytianye@163.com (Y.T.); 2019105004@njau.edu.cn (Y.Z.); 2019205008@njau.edu.cn (W.Y.); melakshaa@yahoo.com (S.M.); 2017205007@njau.edu.cn (Y.N.); wei-wei-4213@njau.edu.cn (W.W.); lifanzhang@njau.edu.cn (L.Z.); 2 Animal Production Research Institute, Agricultural Research Center, Ministry of Agriculture and Land Reclamation, Giza 12618, Egypt

**Keywords:** ACAT2, microenvironment, pre-adipocytes, differentiation, LDLR, SREBP2

## Abstract

Intramuscular fat (IMF) is considered as the fat deposited between muscle fibers. The extracellular matrix microenvironment of adipose tissue is of critical importance for the differentiation, remodeling and function of adipocytes. Therefore, in this study we extracted the muscle tissue centrifugal fluid (MTF) of the longissimus dorsi of Erhualian pigs to mimic the microenvironment of intramuscular pre-adipocytes. MTF of pigs with low intramuscular fat level can inhibit pig intramuscular pre-adipocytes differentiation. Then, proteomics technology (iTRAQ) was used to analyze the MTF with different IMF content, and it was found that individuals with high IMF had low ACAT2 (Acyl-CoA: cholesterol acyltransferases 2) levels, while individuals with low IMF had high ACAT2 levels. Significant changes took place in the pathways involved in coenzyme A, which are closely related to fat and cholesterol metabolism. Therefore, we speculate that ACAT2, as an important element involved in cholesterol metabolism, may become a potential molecular marker for the mechanism of pig intramuscular preadipocytes differentiation. Overexpression of ACAT2 in pig intramuscular pre-adipocytes can inhibit their differentiation, while adding ACAT2 inhibitor avasimibe can rescue the process. Knockdown of srebp2 or ldlr, which are two key genes closely related to ACAT2 and cholesterol metabolism, can inhibit pig intramuscular pre-adipocytes differentiation. Overall, our results suggest that ACAT2 is a novel negative regulator of intramuscular adipocyte differentiation through regulation of pparγ, cebpα signaling and srebp2/ldlr signaling involved in cholesterol metabolism.

## 1. Introduction

Intramuscular fat (IMF), the fat deposited between muscle fibers, has negative effects on health, closely related to insulin resistance and obesity [1]. The extracellular matrix microenvironment of adipose tissue is of critical importance for the differentiation, remodeling and function of adipocytes [2]. Compared with subcutaneous adipocytes, intramuscular adipocytes have lower lipogenic enzyme activities, and lower lipid content [3]. Our previous studies have shown that muscle conditioned medium, as a simulation of the extracellular microenvironment, can reduce intramuscular adipocyte differentiation and lipid accumulation [4,5].

Triglycerides are widely involved in lipid metabolism, and some studies have shown that cholesterol plays an important role in lipid metabolism [6,7]. Cholesterol, a key component of animal cell membranes, functions in cell structure and some signal pathways. Free cholesterol is mainly located in the cell membrane while excessive free cholesterol induces cytotoxicity [8]. Basically, there is a regulatory system for cholesterol homeostasis, which mainly relies on the SREBP/SCAP pathway [9]. SREBP, located in endoplasmic reticulum (ER), is a sensor for cellular free cholesterol [10,11]. When free cholesterol level increases, the SREBP/SCAP pathway can upregulate the expression of enzymes for cholesterol synthesis, such as 3- hydroxy-3-methyl-glutaryl-coenzyme A (HMGCR) and low- density lipoprotein receptor (LDLR) both in post-transcription and after-transcription [12,13].

Besides, the esterification reaction of cholesterol is an important method of cholesterol homeostasis. ACAT1 and ACAT2 are two key genes encoding cholesterol esterification enzymes that convert free cholesterol to cholesteryl esters for storage [14,15]. Both are membrane-spanning proteins located in the ER [16]. ACAT1 is ubiquitously expressed, while ACAT2 is mainly expressed in the liver and small intestine [17]. Cholesteryl esters coalesce in aqueous medium and form cytoplasmic lipid droplets [18]. It is an essential way to protect against the accumulation of free cholesterol within the cell membranes. While a large amount of cholesterol is located in adipocytes, interestingly the expression level of ACATs is low in adipocytes [19,20]. Due to the low expression of ACATs, the main form of stored cholesterol is free form [21,22]. Studies have shown that ACATs over-expressed in mouse cells can inhibit the differentiation of pre-adipocytes, make mature lipid droplets size smaller and increase the free cholesterol level on the surface of the lipid droplets, thereby hindering the function of adipocytes [23]. However, there is very little research on ACATs in pig intramuscular fat cells. Considering the intramuscular adipocytes microenvironment, therefore, we hypothesized that changes in cholesterol homeostasis caused by the enzyme activity of ACAT2 may affect deposition of intramuscular fat.

Proteomics is a developing discipline which studies cell, tissue or body fluid composition and functions of identified proteins. Isobaric tags for relative and absolute quantitation (iTRAQ) are a new technique in comparative proteomic approaches, with the advantages of high sensitivity, accurate quantification and repeatability [24]. Compared with typical 2D electrophoresis, iTRAQ is more reliable. As we know, there is currently no report using iTRAQ to analyze the proteome of porcine IMF. Therefore, iTRAQ has great potential to discover biomarkers associated with the deposition of intramuscular fat.

In this study, we extracted the muscle tissue fluid of Erhualian pig to simulate the microenvironment of intramuscular pre-adipocytes and selected the muscle tissue fluid of individuals with extremely high and low intramuscular fat content for proteomics analysis. The results showed that ACAT2 was significantly differentially expressed in the two groups. We further investigated the effects of increased or decreased ACAT2 in pig pre-adipocytes. Our data demonstrated that increased ACAT2 activity reduced the levels of SREBP2, LDLR, PPARγ and CEBPα, then impaired differentiation of intramuscular pre-adipocytes.

## 2. Materials and Methods

### 2.1. Samples

Eight-month-old female Erhualian Pigs were from Erhualian Seed Conservation Base, Wujin District, Changzhou City, Jiangsu Province, China. All studies were carried out under the NJAU Animal Care Facility guidelines and local ethical approval from Toulouse. The detailed phenotype data were shown in Table 1.

### 2.2. Obtaining Muscle Tissue Fluid

Approximately 10 g of the longissimus dorsi muscle was collected from each sample after slaughter, rinsed with normal saline three times, trimmed in a petri dish to remove excess tissue, and then quickly rinsed three times with normal saline. Put the processed muscle into a net bag after autoclaving, hung it in a 50 mL centrifuge tube containing 5 µL PMSF, centrifuged at 1200 rpm at 4 °C for 15 min, carefully transferred liquid into the cryotube, then quickly stored in liquid nitrogen.

### 2.3. Obtaining Intramuscular Pre-Adipocytes

Seven-day-old Erhualian piglets from the Erhualian Pig Breeding Base (Wujin District, Changzhou, Jiangsu Province, China) were killed by intraperitoneal injection of pentobarbital sodium (50 mg/kg body weight) followed by exsanguinations aseptically. Then use a scalpel and tweezers to carefully lift the back skin to obtain the longissimus dorsi. Under aseptic operating conditions, wash the longissimus dorsi with PBS containing 1% penicillin-streptomycin at 37 °C for 3 times. Cut the longissimus dorsi muscle into a size of about 1 mm^3^ in a disposable petri dish, and transfer to a 50 mL centrifuge tube. Add 2 times the volume of 0.1% type I collagenase digestion solution (containing 1.5% BSA, 1% penicillin-streptomycin), and digest at 37 °C for 1 h with shaking. Add an equal volume of DMEM containing 10% FBS and mix well to terminate the digestion. Use a 200 µm Sieve to filter out undigested tissue mass. Filter again with a 70 µm sieve. Centrifuge at 200× *g* for 10 min at room temperature, carefully aspirate the upper mature adipocytes, add DMEM to wash, resuspend, and centrifuge again. Aspirate the mature adipocytes on the upper surface and transfer it to a 25 cm^2^ culture flask. Fill the culture flask with ordinary culture medium without leaving air bubbles. Invert the culture for 3 days. It can be observed that some mature adipocytes stick to the top of the flask. Around 10 days, a large number of fibrous, dedifferentiated pre-adipocytes were observed and the culture medium was discarded and converted to normal culture. The medium was changed every 2 days until the cells were confluent.

### 2.4. Cell Culture and Differentiation

Primary porcine intramuscular pre-adipocytes were maintained in Dulbecco’s modified Eagle’s medium (DMEM) supplemented with 10% fetal bovine serum (FBS) and 1 % penicillin-streptomycin in 37 °C incubator with 5% CO_2_.

For induction of adipogenic differentiation, cells were first cultured in DMI (DMEM with 10% FBS, 1% penicillin-streptomycin, 2.5 µM dexamethasone, 0.1 mM isobutylmethylxanthine, and 8.6 µM insulin) for three days and then in DMEM with 10% FBS and 8.6 µM insulin until complete differentiation (no more lipid droplets became larger and more). During this period, the culture medium was changed every two days.

### 2.5. Proteomics Analysis

100 µg peptide mixture of each protein sample was labeled using iTRAQ reagent. All operations were carried out following the manufacturer’s protocol.

#### 2.5.1. Protein Extraction

Collected adipocytes or tissues of Erhualian pigs from longissimus dorsi muscle, and muscle tissue fluid. Then homogenized cells or tissues with 1:10 (*w*/*v*) RIPA total protein lysate (Beyotime Biotechnology, Beijing China). After 10 min standing in ice-bath, centrifuged at 12,000 rpm, 4 °C for 20 min, then collected the supernatant. Protein was quantified using BCA Protein Assay Kit (Beyotime Biotechnology, Beijing, China). The quantified protein was stored at -80 °C for proteomics analysis or western blot.

#### 2.5.2. iTRAQ Labeling

After collecting the samples, the follow-up process of proteomics will be implemented by CapitalBio Technology, Beijing.

#### 2.5.3. Liquid Chromatography (LC) and Tandem Mass Spectrometry (MS/MS)

The LC-MS/MS analysis was performed on a Q Exactive mass spectrometer (Thermo Scientific) that was coupled to Easy nLC (Thermo Fisher Scientific, Shanghai, China) for 60/120/240 min. The peptides were loaded onto a reverse phase trap column (Acclaim PepMap100, 100 µm × 2 cm, nanoViper C18, purchased from Thermo Scientific, Shanghai, China) connected to the C18-reversed phase analytical column (Easy Column, 10 cm long, 75 µm inner diameter, 3 µm resin, purchased from Thermo Scientific, Shanghai, China) in buffer A (0.1% Formic acid) and separated with a linear gradient of buffer B (84% acetonitrile and 0.1% Formic acid) at a flow rate of 300 nl/min controlled by IntelliFlow technology. The mass spectrometer was operated in positive ion mode. MS data were acquired using a data-dependent top10 method dynamically choosing the most abundant precursor ions from the survey scan (300–1800 *m*/*z*) for HCD fragmentation. Automatic gain control (AGC) target was set to 3 × 10^6^, and maximum inject time to 10 ms. Dynamic exclusion duration was 40.0 s. Survey scans were acquired at a resolution of 70,000 at *m*/*z* 200 and resolution for HCD spectra was set to 17,500 at *m*/*z* 200, and isolation width was 2 *m*/*z*. Normalized collision energy was 30 eV and the underfill ratio, which specifies the minimum percentage of the target value likely to be reached at maximum fill time, was defined as 0.1%. The instrument was run with peptide recognition mode enabled.

#### 2.5.4. Cluster Analysis of Phosphorylated Peptides

Cluster 3.0 (Stanford, CA, USA; http://bonsai.hgc.jp/~mdehoon/software/cluster/software.htm, 25 December 2017) and Java Treeview software (version 3.0, Boston, MA, USA; http://jtreeview.sourceforge.net, 25 December 2017) were used to performing hierarchical clustering analysis. Euclidean distance algorithm for similarity measure and average linkage clustering algorithm (clustering uses the centroids of the observations) for clustering were selected when performing hierarchical clustering. A heat map was often presented as a visual aid in addition to the dendrogram.

#### 2.5.5. GO Analysis

The protein sequences of the selected differentially expressed proteins were locally searched using the NCBI BLAST+ client software (ncbi-blast-2.2.28+-win32.exe, Beijing, China) and InterProScan to find homologue sequences, then gene ontology (GO) terms were mapped and sequences were annotated using the software program Blast2GO. The GO annotation results were plotted by R scripts.

#### 2.5.6. KEGG Analysis

According to the analysis above, the focused protein was blasted via the online Kyoto Encyclopedia of Genes and Genomes (KEGG) database (http://geneontology.org/, 25 December 2017) to retrieve their KEGG orthology identifications and were subsequently mapped to pathways in KEGG.

#### 2.5.7. Enrichment Analysis

Enrichment analysis was basically based on the Fisher’ exact test, considering the whole quantified proteins as background dataset. Benjamini–Hochberg correction for multiple testing was further applied to adjust derived *p*-values. Only functional categories and pathways with *p*-values under a threshold of 0.05 were considered as significant.

#### 2.5.8. Protein–Protein Interaction Analysis

The protein–protein interaction information of the focused proteins was retrieved from STRING online software (http://string-db.org/, 25 December 2017).

### 2.6. Cell Proliferation Assay

Cells were cultured in 96-well plate, 5000 cell density per well. After 24 h, 48 h, and 72 h of culturing, cells were incubated with 10 µL CCK8 (Vazyme, Nanjing, Jiangsu Province, China) working solution in each plate at 37 °C for 2 h. The absorbance was measured using an automated microplate reader (Bio-Rad, Hercules, CA, USA) at 450 nm after cell incubation. Eight repetitions were set up in each group.

### 2.7. Treatment of ACAT Inhibitor

ACAT inhibitor, Avasimibe (MedChemExpress, Monmouth Junction, NJ, USA) was dissolved in DMEM and formulated into a 20 µM/mL treatment solution. Added this treatment solution into cell for 12 h, then changed the culture.

### 2.8. Transfection of LDLR and SREBP2 siRNAs

The siRNAs were provided by Gene Pharma (Shanghai, China). The sequences of siRNAs were listed in Table 2. The isolated porcine intramuscular pre-adipocytes were cultured in T25 cell culture flasks, 6-well cell plates, or 12-well cell plates for 48 h and then transfected. Before transfection, slowly washed away dead cells and impurities on upper layer with 1×PBS buffer. The transfection reagent Lipofectamine 3000 (Invitrogen, Shanghai, China) provided by Invitrogen was used for transfection with following all operation instructions included in the kit.

### 2.9. Oil Red O Staining and Determination of Triglyceride Content

8 days of differentiated adipocytes were washed 3 times with sterile PBS. Then cells were fixed in 4% paraformaldehyde at 37 °C for 30 min. Washed cells 3 times with PBS, then stained cells with Oil Red O solution for 30 min at room temperature, and washed cells 3 times with PBS. Subsequently, used 60% isopropanol washed cells for 10 s, then observed cells under microscope, taking pictures. An equal volume of isopropanol was added to each well, and after thorough mixing for 1 h, the absorbance value at 510 nm was measured as the content of triglyceride by a full-wavelength microplate reader (Thermo Scientific, Waltham, MA, USA). Each experiment was repeated 3 times.

### 2.10. Triglyceride and Cholesterol Content Detection by Reagent Kit

Cholesterol test kit (A111-1-1) and triglyceride (TG) test kit (A110-1-1) were purchased from Nanjing Jiancheng Institute of Bioengineering, Nanjing, Jiangsu Province, China. The test was carried out in accordance with the instructions.

### 2.11. Total RNA Isolation and RT-PCR Analysis

Total RNA was extracted by using the RNApure kit (BioTeke, Beijing, China). RNA concentrations were determined by using NanoDrop 2000 Spec-trophotometer (Thermo Fisher Scientific, Waltham, MA, USA). Reverse transcription involved 500 ng of RNA by using RT kit according to the manufacturer’s instructions (Takara, Shiga, Japan). The primers pparγ, c/ebpα, ldlr, srebp2 and rplp0 were provided by Qingke Bio Company (Nanjing, Jiangsu province, China). RT-PCR reaction was carried out with 2 µL cDNA samples, 0.5 µL of 10 µM primers and 10 µL SYBR Green Master Mix (Vazyme Biotech, Nanjing, Jiangsu province, China), and 7 µL DEPC water in 96-well plates in Step-one plus (Applied Biosystems, San Francisco, CA, USA). All reactions were performed at 95 °C (10 min), 95 °C (15 s), 60 °C (1 min), then 40 cycles of 95 °C (15 s), 60 °C (30 s), and 95 °C (15 s). The results were analyzed by 2^−ΔΔCt^ method, and all values were normalized to rplp0 mRNA expression level. All RT-PCR primers sequences were listed in Table 3.

### 2.12. Western Blot

Briefly, extracted protein samples from cells were separated by SDS-PAGE, then transferred to nitrocellulose membrane and incubated with the primary antibodies. After incubation with horseradish-peroxidase-conjugated (HPR) secondary antibody for 2 h at room temperature, the protein expression was visualized with BeyoECL Moon Kit (Beyotime, Beijing, China) by Versa DosTM 4000 MP (Bio-Rad Laboratories, Munich, Germany).

Rabbit Anti-SOAT2 antibody (bs-5020R, Biosis, Beijing, China), Rabbit Anti-SREBP2 antibody (bs-2536R, Biosis, Beijing, China), LDL Receptor Rabbit Monoclonal Antibody (AF1438, Beyotime Biotechnology, Shanghai, China), PPAR-γ (I106) polyclonal antibody (BS1587, Bioworld Technology, Inc., MN, USA), CEBP Alpha Rabbit pAb (383901, ZENBIO, Chengdu, Sichuan Province, China), Anti-GAPDH [6C5]—Loading Control (ab8245, Abcam, Cambridge, UK).

### 2.13. Quantification and Statistical Analysis

All data are represented in Mean ± SEM. One-way analysis of variance (ANOVA) and paired sample T-test is used to evaluate statistical significance by SPSS 20.0 (IBM, Armonk, NY, USA). *p*-value of < 0.05 (*), < 0.01 (**), and < 0.001 (***) were considered significant and asterisked in the relevant plots.

## 3. Results

### 3.1. Muscle Tissue Fluid (MTF) of Pigs with Low Intramuscular Fat Level Can Inhibit Pig Intramuscular Pre-Adipocytes Differentiation

**Figure 1:**
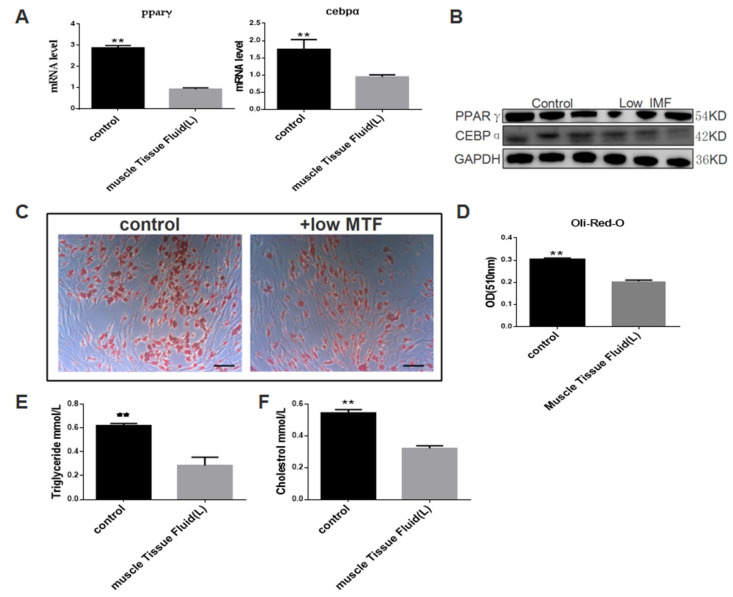
Muscle tissue fluid of pig with low intramuscular fat level can inhibit pig intramuscular preadipocytes differentiation. (**A**) mRNA levels of pparγ and cebpα in the groups of muscle tissue fluid treated, and control. (**B**) protein levels of PPARγ and CEBPα in the groups of muscle tissue fluid treated and control, GAPDH as an internal reference. (**C**) Oil Red O staining in the groups of muscle tissue fluid treated and control. (**D**) Quantification of Oil Red O staining in the groups of muscle tissue fluid treated and control. (**E**) Triglycerides levels in the groups of muscle tissue fluid treated and control. (**F**) Cholesterol levels in the groups of muscle tissue fluid treated and control. Data are expressed as means ± SEM (*n* = 3), representative of three independent experiments. The statistical significance was calculated by One-way ANOVA, ** *p* < 0.01. Scale bar = 100 µm.

To examine the exact role of MTF in intramuscular pre-adipocytes differentiation, we treated pre-adipocytes with MTF (Figure S1). The triglyceride content and cholesterol content in the culture medium of our treatment system were tested and we found no significant differences in the cholesterol and triglyceride content between the culture medium with and without muscle tissue fluid (Figure S2A,B). Then we detected the mRNA and protein expression of pparγ and cebpα and Oil Red O staining to confirm the function of MTF on intramuscular pre-adipocytes differentiation. The muscle tissue fluid of individuals with very high intramuscular fat content had no effect on the differentiation of intramuscular pre-adipocytes (Figure S2C–H). The muscle tissue fluid of individuals with very low intramuscular fat content can reduce the expression levels of pparγ and cebpα (Figure 1A,B). Oil Red O staining, triglyceride content determination and cholesterol content determination results showed that the differentiation of intramuscular pre-adipocytes was inhibited (Figure 1C–F).

### 3.2. iTRAQ Analysis of Muscle Tissue Fluid (MTF)

**Figure 2:**
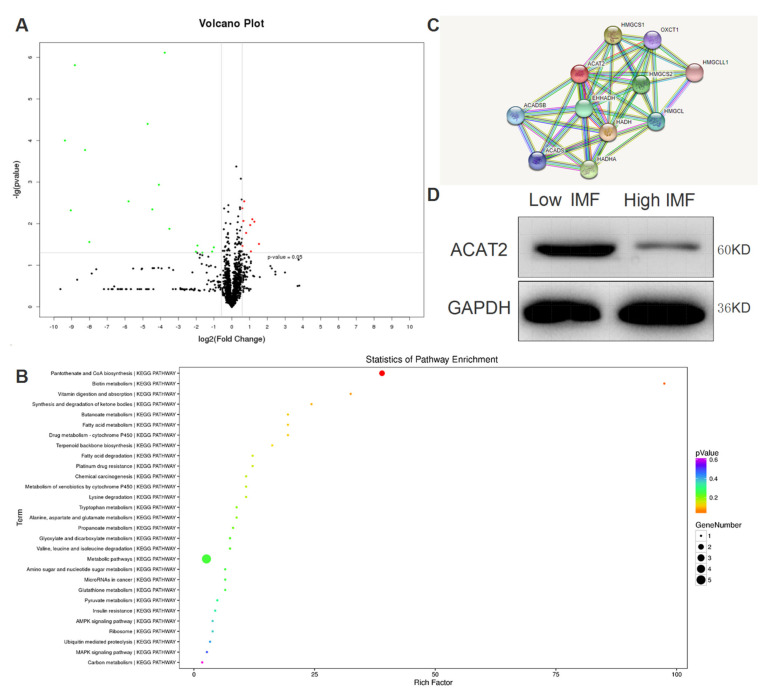
iTRAQ Analysis of Muscle Tissue Fluid. (**A**) Volcano plot shows –log10 ANOVA *p*-values (*y*-axis) plotted against log2 values of protein fold changes (*x*-axis) for quantitative analysis of proteins abundance comparing High and Low groups, classified on intramuscular fat content. Green and red points represent significant (*p*-values < 0.05) up and down -regulated proteins, respectively. (**B**) Picture of KEGG Pathway. Differentially up-regulated proteins are marked with a red background, and differentially down-regulated proteins are marked with a blue background. The number in the box represents enzyme, indicating that the corresponding protein is related to this enzyme, and the entire signal pathway is composed of many different enzymes through complex biochemical reactions. (**C**) Network of predicted protein–protein interactions against database from differentially abundant proteins between high and low groups based on intramuscular fat content. (**D**) Western-blot verification of significantly differentially expressed protein ACAT2, GAPDH as an internal reference.

The high (H) and low (L) extreme intramuscular fat content of three Erhualian pigs was reported in Table 1 and muscle tissue centrifuged fluids were obtained separately for further iTRAQ analysis. Based on the porcine protein database, the raw data of mass spectrometry were analyzed separately. The raw data for mass detection are generated by Proteome Discoverer software (called PD software) using the Sequest algorithm in the above database for search and calculation. Trypsin-specific digestion allows up to two missing cleavage sites. Cys iodoacetylation and iTRAQ modification were set as fixed modification parameters, and methionine oxidation and phosphorylation were set as variable modification parameters. The tolerance is 15 ppm, and the product ion mass tolerance is 0.02 Da. Select highly reliable peptides at 1% FDR as the filtering parameters for qualitative protein identification, and select specific peptides for relative quantitative analysis of proteins between different samples. A total of 1671 proteins were identified, of which 10 were significantly up-regulated and 16 were significantly down-regulated (Figure S3). The GO analysis and KEGG analysis of proteome data showed that the individual’s metabolism pathway changed significantly, and the synthesis pathway of pantothenic acid and coenzyme A changed significantly (Figure 2A–C). Among them, ACAT2 is an extremely significant down-regulated protein in individuals with high intramuscular fat (Figure 2D), and it is an important factor involved in cholesterol metabolism and coenzyme A synthesis pathway, which may become a new molecular marker for fat deposition in muscle.

### 3.3. Muscle Tissue Fluid of Pig with Low Intramuscular Fat Level Can Affects srebp2/ldlr Level of Adipocytes

**Figure 3:**
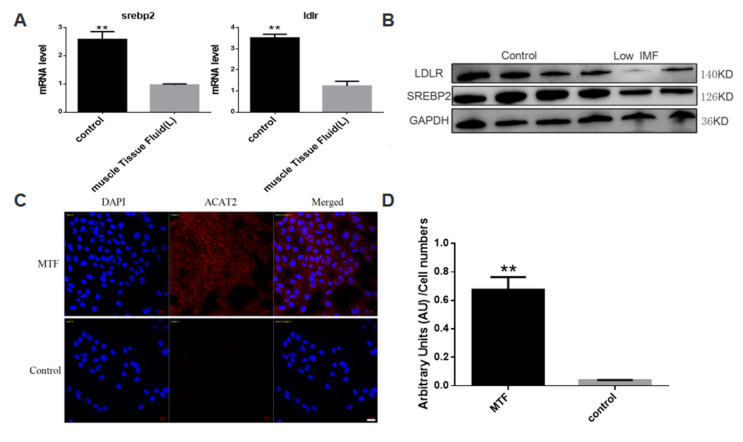
Muscle tissue fluid of pig with low intramuscular fat level can affects srebp2/ldlr level of adipocytes. (**A**) mRNA levels of SREBP2 and LDLR in the groups of muscle tissue fluid treated and control. (**B**) protein levels of SREBP2 and LDLR in the groups of muscle tissue fluid treated and control, GAPDH as an internal reference. (**C**) pig intramuscular preadipocytes treated with muscle tissue fluid of low intramuscular fat individual for 12 h. Representative images by confocal microscopy are presented (*n* = 3). (**D**) Fluorescence intensity of pig intramuscular preadipocytes treated with muscle tissue fluid of low intramuscular fat individual for 12 h, quantified by IntDen/Cell Numbers (*n* = 3). Data are expressed as means ± SEM (*n* = 3), representative of 3 independent experiments. The statistical significance was calculated by One-way ANOVA, ** *p* < 0.01. Scale bar = 20 µm.

According to the result of iTRAQ analysis, muscle tissue fluid of individuals with very low IMF content contains a higher concentration of ACAT2. Since normal adipose tissue maintains very low ACAT2 expression and activity [19,20], and ACAT2 is an important factor involved in cholesterol metabolism, we suspect that excessively high level of ACAT2 will cause the destruction of cholesterol homeostasis in intramuscular adipocytes, thereby inhibiting the differentiation of intramuscular pre-adipocytes. Our proteomic analysis results suggest that cholesterol metabolism levels are different among individuals with different IMF levels. SREBP2 and LDLR are two key regulators of cholesterol metabolism, so we detected the mRNA expression and protein levels of SREBP2 and LDLR; the results showed that after adding muscle tissue fluid containing high concentration of ACAT2 (Figure 3C,D), the expression levels of SREBP2 and LDLR were inhibited (Figure 3A,B). Therefore, we speculate that high concentration of ACAT2 in muscle tissue fluid inhibits intramuscular pre-adipocytes differentiation through cholesterol metabolism pathway.

### 3.4. Overexpression of ACAT2 in Pig Intramuscular Pre-Adipocytes Can Inhibit Its Differentiation, Adding ACAT Inhibitor Avasimibe Can Rescue the Process

**Figure 4:**
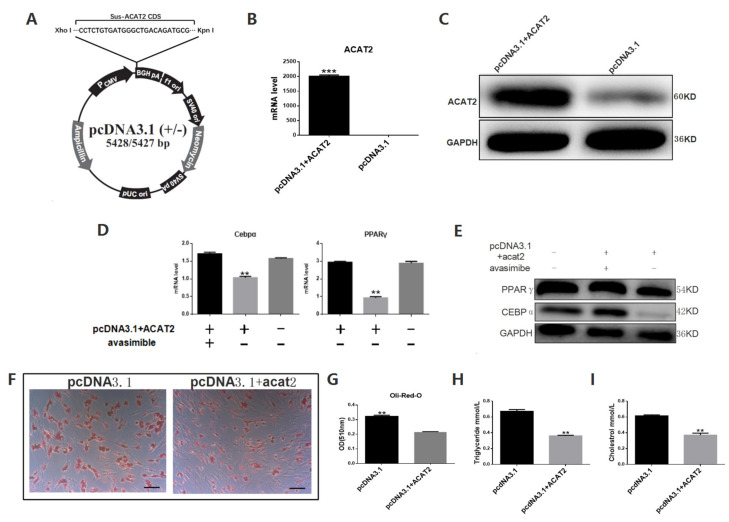
Overexpression of ACAT2 in pig intramuscular preadipocytes can inhibit its differentiation, adding ACAT inhibitor avasimibe can rescue the process. (**A**) Construction of ACAT2 Overexpression Vector. (**B**) The efficiency of overexpression was detected in mRNA level. (**C**) The efficiency of overexpression was detected in protein level. (**D**) mRNA levels of pparγ and cebpα in the groups of pcDNA3.1-acat2 and avasimibe both treated, only pcDNA-acat2 treated, and pcDNA3.1. (**E**) Protein levels of PPARγ and CEBPα in the groups of pcDNA3.1-acat2 and avasimibe both treated, only pcDNA-acat2 treated, and pcDNA3.1. (**F**) Oil Red O staining in the groups of pcDNA3.1-acat2 treated and control. (**G**) Quantification of Oil Red O staining in the groups of pcDNA3.1-acat2 treated and control. (**H**) Triglycerides levels in the groups of pcDNA3.1 and pcDNA-acat2 treated. (**I**) Cholesterol levels in the groups of pcDNA3.1 and pcDNA-acat2 treated. Data are expressed as means ± SEM (*n* = 3), representative of 3 independent experiments. The statistical significance was calculated by One-way ANOVA, ** *p* < 0.01, *** *p* < 0.001. Scale bar = 100 µm.

In order to verify that high concentration of ACAT2 can inhibit pig intramuscular pre-adipocytes differentiation, we transferred the plasmid overexpressing ACAT2 into pig intramuscular pre-adipocytes (Figure 4A–C). We found that after overexpressing ACAT2, the mRNA expression and protein levels of pparγ and cebpα in pig intramuscular pre-adipocytes were reduced (Figure 4D,E), combined with the results of Oil Red O staining, triglyceride content determination and cholesterol content determination (Figure 4F–I), showed that the differentiation of pig intramuscular pre-adipocytes were inhibited. After adding the ACAT inhibitor avasimibe, the expression levels of pparγ and cebpα recovered (Figure 4D,E) Therefore, we found that an excessively high level of ACAT2 inhibits the pig intramuscular pre-adipocytes differentiation.

### 3.5. Blocking the srebp2/ldlr Signal Can Inhibit Pig Intramuscular Pre-Adipocytes Differentiation, and the Effect of ACAT2 Disappears

**Figure 5:**
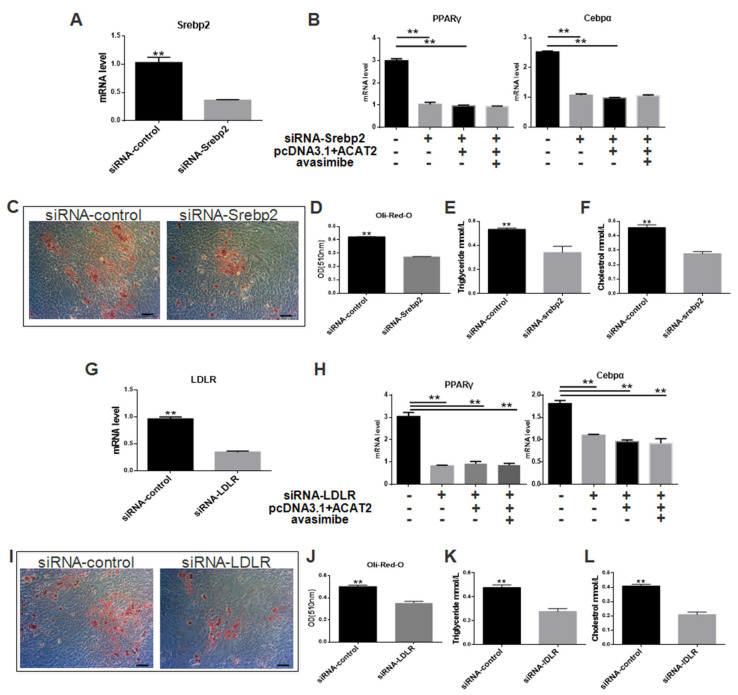
Blocking srebp2/ldlr signal can inhibit pig intramuscular pre-adipocytes differentiation, and the effect of ACAT2 disappears. (**A**) The interfering efficiency of siRNA-Srebp2. (**B**) mRNA levels of pparγ and cebpα in different treatment groups. (**C**) Oil Red O staining in the groups of siRNA-control and siRNA-srebp2. (**D**) Quantification Oil Red O staining in the groups of siRNA-control and siRNA-srebp2. (**E**) Triglycerides levels in the groups of siRNA-control and siRNA-srebp2. (**F**) Cholesterol levels in the groups of siRNA-control and siRNA-srebp2. (**G**) The interfering efficiency of siRNA-ldlr. (**H**) mRNA levels of pparγ and cebpα in different treatment groups. (**I**) Oil Red O staining in the groups of siRNA-control and siRNA-ldlr. (**J**) Quantification Oil Red O staining in the groups of siRNA-control and siRNA-ldlr. (**K**) Triglycerides levels in the groups of siRNA-control and siRNA-ldlr. (**L**) Cholesterol levels in the groups of siRNA-control and siRNA-ldlr. Data are expressed as means ± SEM (*n* = 3), representative of 3 independent experiments. The statistical significance was calculated by One-way ANOVA, ** *p* < 0.01.

In order to explore the mechanism of ACAT2 regulating pig fat deposition, we synthesized siRNAs for two key factors in the cholesterol metabolism pathway, srebp2 and ldlr, and knocked down these two factors, respectively (Figure 5A,G). After knocking down srebp2, the differentiation of pig intramuscular pre-adipocytes was inhibited (Figure 5C–F), the expression levels of pparγ and cebpα were reduced (Figure 5B). In cells co-transfected with siRNA-srebp2 and the plasmid overexpressing ACAT2 was not responded after knocking down srebp2, adding the ACAT2 inhibitor avasimibe had no effect (Figure 5B). After knocking down ldlr, the differentiation of pig intramuscular pre-adipocytes was inhibited (Figure 5I–L), the expression levels of pparγ and cebpα decreased (Figure 5H). In the cells co-transfected with siRNA-ldlr and the plasmid overexpressing acat2 was not responded after knocking down ldlr, adding the ACAT inhibitor avasimibe had no effect (Figure 5H). ACAT2 did not function well when the SREBP2/LDLR pathway is disrupted.

## 4. Discussion

Due to the special deposition location, it is interesting to study the mechanism of how microenvironment affects intramuscular fat (IMF) deposition. The microenvironment of fat from different positions regulates the metabolism of fat cells. In the 3D-ECM-adipocyte culture, the microenvironment of the subcutaneous fat tissue of the inguinal enhances the uptake of glucose in the fat cells of the visceral fat tissue due to high-fat feeding, while the microenvironment of visceral fat can impair the expression of adipogenic genes in the fat cells of subcutaneous fat [25]. In the tumor environment, fat cells may also undergo remodeling. Adipose tissue interacts with tumors. Tumors will put pressure on adipose tissue for the purpose of its own evolution. The remodeled adipocytes by tumors increase the secretion of a variety of inflammatory cytokines, chemokines, and adipokines [26]. Interestingly, tumors can also cause physical stress on adipocytes and induce adipocytes to dedifferentiate into a state similar to MSCs, called compression-induced adipocyte dedifferentiation, which is characterized by the ability to form clones and differentiate into multiple lines [27]. Therefore, in order to study the influence of the special microenvironment of intramuscular fat on the differentiation of intramuscular fat cells, we extracted muscle tissue centrifugal fluid to imitate the microenvironment of intramuscular fat and performed iTRAQ analysis to find the key factors and pathways that can affect the differentiation of intramuscular fat. According to our results, ACAT2 and cholesterol pathways can be the candidates object for further research.

Cholesterol plays essential structural and signaling roles in mammalian cells, and adipose tissue can store above 50% of whole-body cholesterol [28,29]. However, there are few studies on the mechanism of fat deposition by cholesterol pathway. Previous studies have shown that ldlr−/− mice lack adipose tissue and have a higher plasma cholesterol level than wild-type [30]. Therefore, the changes in cholesterol homeostasis caused by the LDLR pathway may lead to reduced adipose tissue. The intracellular cholesterol level of adipose tissue is positively correlated with the size of fat cells and the level of triglycerides [28]. Due to the different deposition locations, the fat storage and differentiation of IMF and subcutaneous fat are different [2]. Compared with subcutaneous fat, IMF has lower lipase activity and lower triglyceride levels. The reason for this difference may come from the microenvironment in which IMF is located. Therefore, we hypothesized that the deposition of intramuscular fat may be related to the cholesterol homeostasis involved in LDLR pathway. Indeed, we show here for the first time that ACAT2 is up-regulated in the pig with low intramuscular fat content (Figure 2). Cholesterol in cells is usually stored as cholesterol ester under the esterification of ACAT. It is interesting that although a large amount of cholesterol is stored in fat cells, the gene expression level of ACAT is extremely low [19,20]. As a cholesterol esterase, ACAT2’s tissue expression is strictly restricted, and it is only expressed in large quantities in the liver and small intestine, which may be due to its stronger effect than ACAT1 [31,32,33]. Excessive ACATs levels can inhibit the differentiation of pre-adipocytes, make mature fat droplets smaller and increase the free cholesterol level on the surface of the lipid droplets [23], at the same time, ACAT2 is down-regulated during adipocyte differentiation [34]. Thus, it is important to determine whether and how increased ACAT2 activity may negatively impact intramuscular fat function. In this work, we demonstrated that the overexpression of ACAT2 impaired intramuscular pre-adipocytes differentiation (Figure 4). We further proved that blocking srebp2/ldlr signal could inhibit pig intramuscular pre-adipocytes differentiation, and the effect of ACAT2 disappeared (Figure 5). SREBP2/LDLR is an important cholesterol signaling pathway. After blocking SREBP2/LDLR, the increase or decrease of ACAT2 activity did not bring any significant changes to intramuscular pre-adipocytes. This may indicate that SREBP2/LDLR signaling pathway plays an important role in ACAT2 functioning, while the relationship among them which needs to be further studied.

In order to explore the mechanism of the different levels of ACAT2 in individuals with different intramuscular fat content in our iTRAQ results. We extracted the DNA of individuals with different intramuscular fat content and analyzed the regulatory sequence of ACAT2. However, no significant results were found (not shown in the paper). As it is reported, Cys277 of ACAT2 can be ubiquitinated for degradation, when the level of certain lipids is low, free cholesterol and saturated free fatty acids can protect the protein from degradation through inducing cellular reactive oxygen species to oxidize Cys277 [34]. This may be the mechanism of ACAT2 individual variation, and it explains that the ubiquitination regulation of ACAT2 may be the reason that adipose tissue, as an important organ for lipid and cholesterol storage, expresses low levels of ACAT2.

In summary, we found a new potential molecular marker ACAT2 related to intramuscular fat deposition. The study of the role of ACAT2 on adipocyte provides a theoretical basis for the mechanism of cholesterol metabolism affecting fat deposition. Our work uncovers a unique aspect in the regulation of cholesterol homeostasis in adipocytes and suggests that increased ACAT2 proteins can inhibit intramuscular pre-adipocyte differentiation. Therefore, ACAT2 inhibitors may be tested in pork quality improvement, body fat distribution research and obesity research.

## 5. Conclusions

Our research proves that ACAT2 with high protein levels inhibits the level of LDLR and SREBP2, destroys the homeostasis of cholesterol metabolism, and thus inhibits pig intramuscular pre-adipocytes differentiation.

## Figures and Tables

**Table 1:** Phenotypic data of selected two groups of extreme intramuscular fat content of Erhualian pigs.

Group	Individual Tags	Weight(kg)	IMF(%)
IMF(H)	47	84	6.692601
	574	83	6.836052
	583	84	7.129091
Average		83.7	6.885915
IMF(L)	882	83	2.912024
	71	83.5	3.393526
	173	82.5	3.29889
Average		83	3.29889

**Table 2:** The information of oligonucleotide.

Oligonucleotide	Oligonucleotide Sequence(5′ to 3′)
NC-siRNA	F: GCG ACG AUC UGC CUA AGA UTT R: AUC UUA GGC AGA UCG UCG CTT
ldlr-siRNA	F: GCU GCA GUU UGU CAG CAA UTT R: AUU GCU GAC AAA CUG CAG CTT
Srebp2-siRNA	F: GGA AAU GCA UCU CCU ACA ATT R: UUG UAG GAG AUG CAU UUC CTT

**Table 3:** Primer sequences used for real-time PCR.

Gene Symbol	Forward Primer (5′ to 3′)	Reverse Primer (5′ to 3′)	Product Sizev (bp)	TM (°C)
pparγ	TTG CTG TGA AGT TCA ACG CA	GTG GTT CAA CTT GAG CTG CA	167	60
rplp0	TCC AGG CTT TAG GCA TCA CC	GGC TCC CAC TTT GTC TCC AG	95	60
c/ebpα	GGC ATC TGC GAA CAC GAG A	AGG AAC TCG TCG TTG AAG GC	74	60
ldlr	GTG TCA ACC GCT GCA TTC C	TGC TTC ATC CGA GCC GTC	191	60
srebp2	ATC ATC AAA ACC GAT TCC CTT	CCT GCT TAA TGG GCA CTT TC	208	60

## Data Availability

All data included in this study are available upon request by contact with the corresponding author.

## Supplementary Materials

The following supporting information can be downloaded at: https://www.mdpi.com/article/10.3390/biom12020237/s1, Figure S1: The effects of MTF on preadipocytes proliferation; Figure S2: Muscle tissue fluid of pig with high intramuscular fat level had no effect on pig intramuscular preadipocytes differentiation; Figure S3: Data detected by proteomic analysis.

## Abbreviation

IMF	Intramuscular fat
MTF	Muscle Tissue Fluid
TG	Triglyceride
DEX	Dexamethasone
INS	Insulin
IBMX	3-isobutyl-1-methylxanthine
PPARγ	Peroxisome proliferator activated receptor γ
CEBPα	CCAAT Enhancer Binding Protein Alpha
RPLP0	Ribosomal protein, large, P0
ACAT2	Acetyl-CoA acetyltransferase, cytosolic
LDLR	Low Density Lipoprotein Receptor
SREBP2	Sterol Regulatory Element Binding Protein 2
BSA	Bovine serum albumin
FBS	Fetal bovine serum
