# Supplementary material for: ACAT2 Is a Novel Negative Regulator of Pig Intramuscular Preadipocytes Differentiation
Source: Biomolecules. 2022 Jan 31;12(2):237. doi: 10.3390/biom12020237 (PMC8961576; doi:10.3390/biom12020237)
Supplement: Supplementary file 1 [file biomolecules-12-00237-s001.zip › biomolecules-1547436-supplementary.pdf]

Supplementary Material

1 Figure S1. The effects of MTF on preadipocytes proliferation

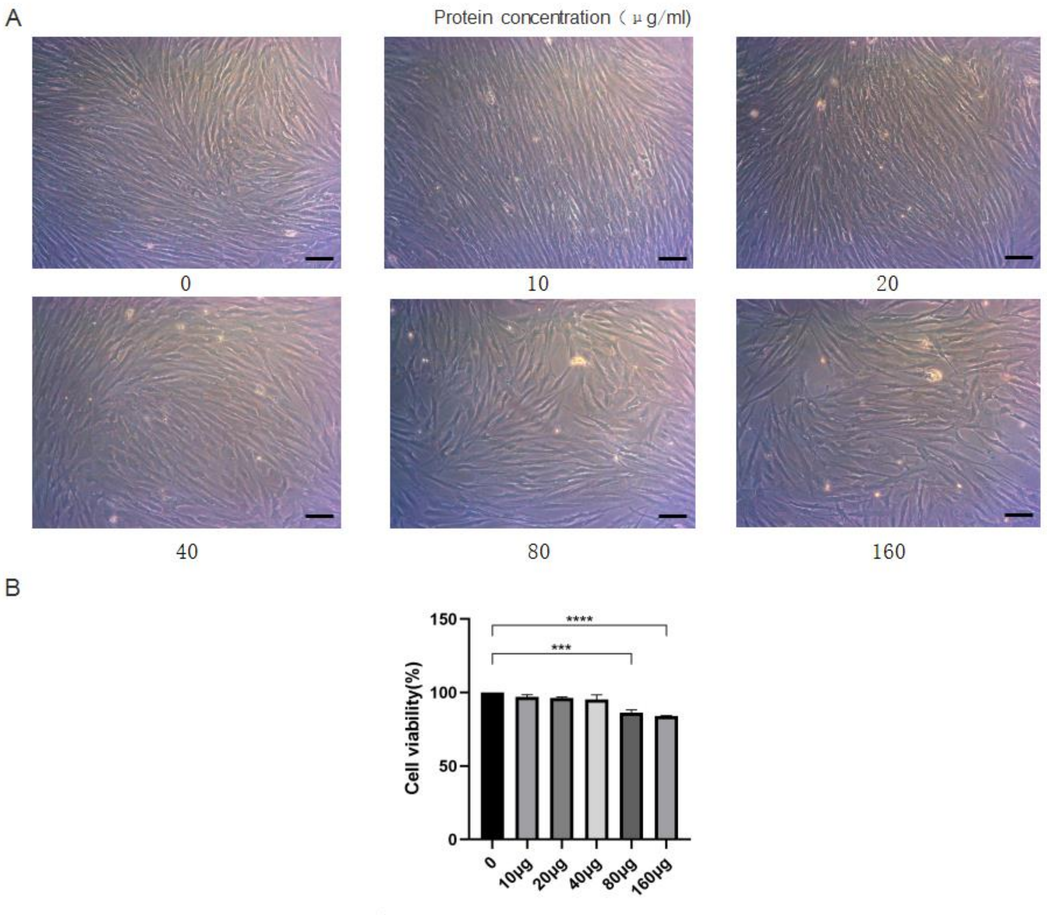

Figure. S1 The effects of MTF on intramuscular preadipocytes proliferation.

A.Photographs of intramuscular preadipocytes treated 2 days with MTF versus physiological saline (A, n = 3). B.The cell viability of preadipocytes after cultured in MTF supplemented medium for 3 days. (B, n = 6)

2 Figure S2. Muscle tissue fluid of pig with high intramuscular fat level had no effect on pig intramuscular preadipocytes differentiation

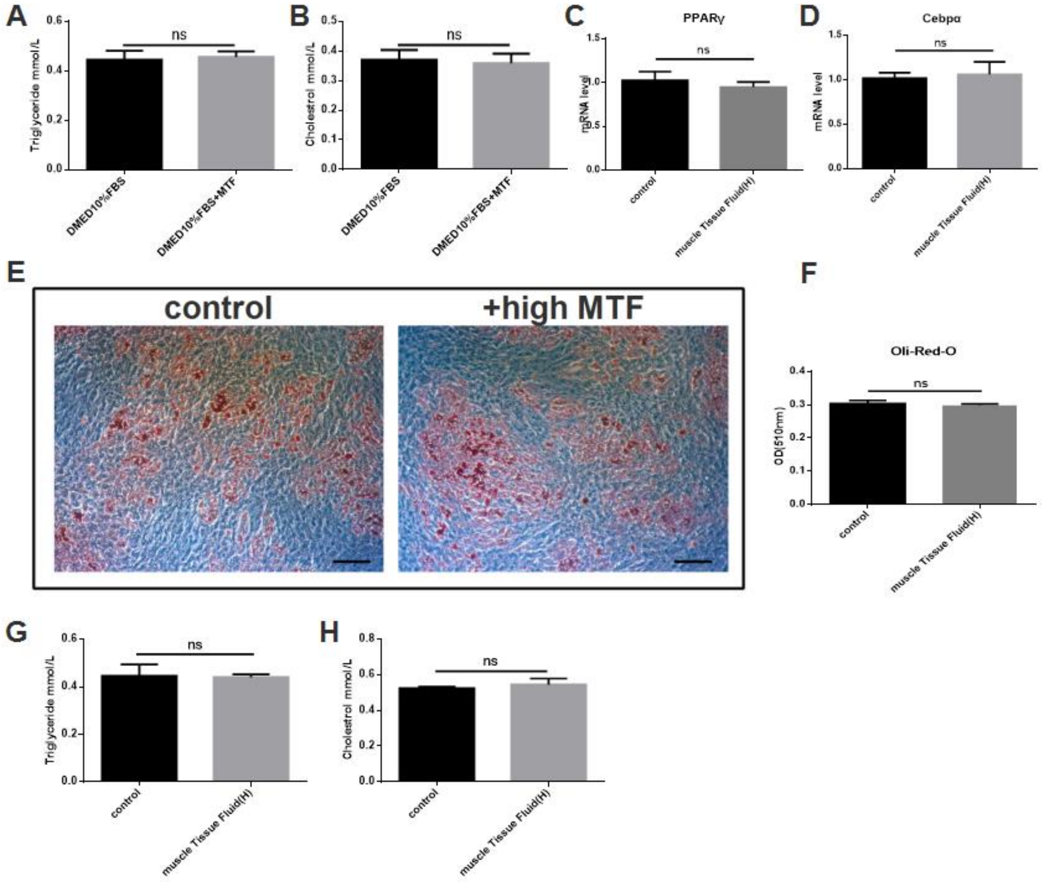

Figure S2. Muscle tissue fluid of pig with high intramuscular fat level had no effect on pig intramuscular preadipocytes differentiation

(A) Triglycerides levels of the normal medium (DMEM10%FBS) and the normal medium (DMEM10%FBS) with MTF. (B) Cholesterol levels of the normal medium (DMEM10%FBS) and the normal medium (DMEM10%FBS) with MTF. (C) mRNA levels of pparγ in the groups of muscle tissue fluid treated and control. (D) mRNA levels of ebpα in the groups of muscle tissue fluid treated and control. (E) Oil Red O staining in the groups of muscle tissue fluid treated and control. (F) Quantification of Oil Red O staining in the groups of muscle tissue fluid treated and control. (G)Triglycerides levels in the groups of muscle tissue fluid treated and control. (H)Cholesterol levels in the groups of muscle tissue fluid treated and control. Data are expressed as means±SEM (n=3), representative of 3 independent experiments. The

statistical significance was calculated by One-way ANOVA *P<0.05, **P<0.01. Scale bar = 100μm.

3 Figure S3. Data detected by proteomic analysis

<table>
<tr><td>Number of proteins identified</td><td colspan="2">1671</td></tr>
<tr><td>Quantifiable protein number</td><td colspan="2">1511</td></tr>
<tr><td>Number of peptides identified</td><td colspan="2">11366</td></tr>
<tr><td>Detection times of peptides</td><td colspan="2">61690</td></tr>
<tr><td>Groups</td><td>Number of proteins up-regulated</td><td>Number of proteins down-regulated</td></tr>
<tr><td>IMF(H)-vs-IMF(L)</td><td>10</td><td>16</td></tr>
</table>
